# Sex-Specific Associations between Gut Prevotellaceae and Host Genetics on Adiposity

**DOI:** 10.3390/microorganisms8060938

**Published:** 2020-06-22

**Authors:** Amanda Cuevas-Sierra, José Ignacio Riezu-Boj, Elizabeth Guruceaga, Fermín Ignacio Milagro, José Alfredo Martínez

**Affiliations:** 1 Department of Nutrition, Food Science, and Physiology, and Center for Nutrition Research, University of Navarra, 31008 Pamplona, Spain; acuevas.1@alumni.unav.es (A.C.-S.); jiriezu@unav.es (J.I.R.-B.); jalfmtz@unav.es (J.A.M.); 2 Navarra Institute for Health Research (IdiSNA), 31008 Pamplona, Spain; eguruce@unav.es; 3 Proteomics, Genomics and Bioinformatics Core Facility, Center for Applied Medical Research, University of Navarra, 31008 Pamplona, Spain; 4 Centro de Investigacion Biomedica en Red Fisiopatología de la Obesidad y Nutricion (CIBERobn), Instituto de Salud Carlos III, 28029 Madrid, Spain

**Keywords:** genetic risk score, gut microbiome, obesity, nutrigenetics, metagenomics

## Abstract

The gut microbiome has been recognized as a tool for understanding adiposity accumulation and for providing personalized nutrition advice for the management of obesity and accompanying metabolic complications. The genetic background is also involved in human energy homeostasis. In order to increase the value of nutrigenetic dietary advice, the interplay between genetics and microbiota must be investigated. The purpose of the present study was to evaluate interactive associations between gut microbiota composition and 95 obesity-related single nucleotide polymorphisms (SNPs) searched in the literature. Oral mucosa and fecal samples from 360 normal weight, overweight and obese subjects were collected. Next generation genotyping of these 95 SNPs and fecal 16S rRNA sequencing were performed. A genetic risk score (GRS) was constructed with 10 SNPs statistically or marginally associated with body mass index (BMI). Several microbiome statistical analyses at family taxonomic level were applied (LEfSe, Canonical Correspondence Analysis, MetagenomeSeq and Random Forest), and Prevotellaceae family was found in all of them as one of the most important bacterial families associated with BMI and GRS. Thus, in this family it was further analyzed the interactive association between BMI and GRS with linear regression models. Interestingly, women with higher abundance of Prevotellaceae and higher GRS were more obese, compared to women with higher GRS and lower abundance of Prevotellaceae. These findings suggest relevant interrelationships between Prevotellaceae and the genetic background that may determine interindividual BMI differences in women, which opens the way to new precision nutrition-based treatments for obesity.

## 1. Introduction

Obesity prevalence is rising dramatically around the world, which is associated with important public health burdens [1,2]. In this context, it has been largely demonstrated that obese subjects have a different gut microbiota composition than lean subjects [3]. Genome-wide association studies in humans [4,5] have identified multiple loci that contribute to obesity and its associated metabolic abnormalities. A major challenge in the field is deciphering how host genetics and gut microbiome interact and the role of this interplay in the context of obesity [6]. Several quantitative trait locus analyses in mice have recognized genetic regions associated with the abundance of several bacterial taxa and community structure [7]. Genome-wide association studies in human twins have already identified heritable bacterial taxa and single nucleotide polymorphisms (SNPs) associated with specific gut microbes. In fact, gut microbiomes of monozygotic twins have been reported to be more similar than those of dizygotic twins, suggesting a potential association between genetic background and gut microbiome components [8,9]. In addition, some trials in mice revealed that the removal of most of the gut microbiota with broad-spectrum antibiotics effectively reduced the development of adiposity in genetically obese mice, showing that these two factors are interplaying in the development of obesity disease [4,5]. Further research in humans have described that some SNPs are associated with the abundance of specific microbial taxa. For example, human variants near the lactase gene have been linked with the abundance of *Bifidobacteria* [10,11], while the genus *Akkermansia* has been associated with a variant near a gene (*PLD1*) that has been previously implicated in body mass index (BMI) [12]. In addition, certain fundamental aspects of metabolic homeostasis are regulated differently in males and females [13]. Interestingly, some studies have recently found that gut microbiota composition may differ between sexes and that these differences may be influenced by the severity of obesity [14,15].

Although some studies have showed that some gene variants (such as FTO) [5] are associated with the abundance of certain bacterial taxa, very few individual microbe-polymorphism associations have been identified that have reached genome-wide significance. Consequently, the role of gut microbiota in genetically predisposed obesity in humans has not been adequately characterized so far [16]. To determine the relative impact of these obesity factors, their hierarchy and interactions is a challenging task. The understanding of the complex network of factors related to obesity, such as host genetic and gut microbiome, could be the key for designing new strategies targeted to achieve a personalized nutrition for obesity patients based on genetic background and gut microbiome composition [17].

The current study aimed to provide novel data and characterize potential interactive associations between the gut microbiome and the human host genetics in relation to BMI as a marker of adiposity, as well as the eventual role of sex in this interplay.

## 2. Materials and Methods 

### 2.1. Study Participants

The current prospective study encompassed baseline data of 296 Spanish subjects from the OBEKIT study (reg. No. NCT02737267, clinicaltrials.gov), who were recruited for a weight loss nutritional intervention at the Center for Nutrition Research of the University of Navarra in the city of Pamplona (Navarra, Spain) as well as 64 normal weight subjects as a control group. All participants self-reported Caucasian ethnicity. The major exclusion criteria were a previous history of cardiovascular disease, diabetes mellitus, and hypertension; women who were pregnant or lactating; reports of weight change within the 3 months before the study; and medication use for weight control and hyperlipidemia management. All research procedures were implemented in accordance with the ethical principles of the 2013 Declaration of Helsinki [18]. All participants voluntarily provided written informed consent before entry into the study. The study protocol was approved by the Research Ethics Committee of the University of Navarra on March 2016 (Ref. 132/2015).

### 2.2. Anthropometric Measurements

Anthropometric measurements, including weight, height, waist and hip circumference, were collected at the beginning of the study by trained nutritionists using conventional validated procedures [19]. BMI was calculated as the ratio between body weight and squared height (kg/m^2^) and the BMI classification criteria was following according to the World Health Organization (normalweight BMI < 24.9 kg/m^2^; overweight BMI < 29.9 kg/m^2^; obese BMI > 30 kg/m^2^) [20]. The percentage of body fat was assessed by bioelectrical impedance using the BC-418 Segmental Body Composition Analyzer (Tanita, Tokyo, Japan). Both systolic and diastolic blood pressures were measured with a sphygmomanometer using the standardized criteria of the World Health Organization and the International Society of Hypertension [21]. 

### 2.3. Habitual Dietary Intake and Physical Activity

Habitual dietary intake at baseline was collected with a validated food frequency questionnaire that included 137 food items with corresponding portion sizes as described elsewhere [22]. All enrolled participants were asked to provide information about the number of times they had consumed each food item during the previous year according to four frequency categories: daily, weekly, monthly, or never. Total energy (kcal) and macronutrient intakes (%) were determined with an ad hoc computer program that was specifically developed for this purpose, by calculating it as the sum of frequency of consumption multiplied by nutrient composition of a specified portion size available from valid Spanish food composition tables [23,24]. Physical activity at baseline was estimated using a previously validated 17-item questionnaire expressed in metabolic equivalents (METS), as detailed elsewhere [25]. 

### 2.4. Biochemical Measurements 

Venous blood samples were drawn by venipuncture after a 12 h overnight fast in a clinical setting. Blood tests were conducted with an automatized analyzer Pentra C200 and suitable kits were provided by the company (HORIBA Medical, Madrid, Spain). The following biochemical markers were assessed in the blood samples: glucose, total cholesterol, high-density lipoprotein cholesterol (HDL-c), triglycerides, alanine-aminotransferase (ALT), aspartate-aminotransferase (AST) and uric acid. Low-density lipoprotein cholesterol (LDL-c) was estimated using the Friedewald equation (LDL-c=TC−HDL-c−triglycerides/5) [26]. Homeostatic model assessment for insulin resistance (HOMA-IR) was calculated using fasting insulin and glucose concentrations [27]. Adiponectin, insulin, leptin, C-reactive protein (CRP), thyroid-stimulating hormone (TSH) and tumor necrosis factor alpha (TNFα) were measured using specific enzyme-linked immunosorbent assays and read with an automated analyzer system (Triturus, Grifols, Barcelona, Spain). The following kits were used: insulin (Mercodia, Uppsala, Sweden), TNFα (R&D Systems, Minneapolis, MN, USA), CRP (Demeditec, Kiel, Germany), adiponectin (BioVendor, Brno, Czech Republic), leptin (Mercodia, Uppsala, Sweden), TSH (Demeditec, Kiel, Germany) following the instructions provided by the manufacturers. 

### 2.5. Fecal Sample Collection and Metagenomic Data

Volunteers self-collected fecal samples at baseline using OMNIgene.GUT kits from DNA Genotek (Ottawa, ON, Canada), according to the standard guidelines provided by the supplier. The isolation of DNA from fecal samples was performed with the QIAamp^®^ DNA kit (Qiagen, Hilden, Germany) following the manufacturer’s protocol. Bacterial DNA sequencing was performed by the Servei de Genòmica i Bioinformàtica (Autonomous University of Barcelona, Barcelona, Spain). The Illumina 16S protocol was followed, which is based on the amplification of the V3-V4 variable regions of the 16S rRNA gene. Paired-end sequencing was performed in the MiSeq System (Illumina, San Diego, CA, USA). In the process, two PCR reactions were carried out. In the first one, 12.5 ng of genomic DNA and the 16S-F and 16S-R primers were used (16S Amplicon PCR Forward Primer =5′ TCGTCGGCAGCGTCAGATGTGTATAAGAGACAGCCTACGGGNGGCWGCAG; 16S Amplicon PCR Reverse Primer = 5′ GTCTCGTGGGCTCGGAGATGTGTATAAGAGACAGGACTACHVGGGTATCTAATCC). The protocol in this first PCR was 95 °C for 3 min and 25 cycles of: 95 °C for 30 s, 55 °C for 30 s, 72 °C for 30 s. Finally, 72 °C for 5 min and hold at 4 °C. Five microliters of the first PCR was used in the second PCR, after the cleaning process. The primers used in this PCR were part of the Nextera^®^ XT DNA Index Kit (96 indexes, 384 samples) FC-131-1002 (Illumina). The protocol for the second PCR was 95 °C for 3 min, 8 cycles of: 95 °C for 30 s, 55 °C for 30 s, 72 °C for 30 s. Finally, 72 °C for 5 min and hold at 4 °C. After each PCR, the quality of the process was checked in a Labchip Bioanalyzer (Agilent Technologies Inc, Santa Clara, CA, USA). Once all the samples were obtained, up to 40 samples were multiplexed in each run of 2 × 300 cycles. For this purpose, equimolar concentrations of each of the samples were mixed and the pool diluted up to 20 pM. A total of 3 runs were performed on the MiSeq sequencer with the MiSeq^®^ Reagent Kit v3 (600 cycle) MS-102-3003. Negative controls were always used and included in each run. Samples were randomized by sex, age and category of obesity/no obesity in order to avoid the batch effect. Adapters and barcodes were removed following the standard Illumina methods. The maximum of reads obtained was 3,986,821 and the minimum 102 (mean = 257,499.8; SD = 268,612.5). Those samples that correspond to good quality readings not obtained at a sequence depth of 40,000 readings were discarded. In total, 4 samples of a total of 364 sequenced samples (1.1%) were removed, resulting 360 samples. Resequencing was performed but the required depth was not obtained. The 16S rRNA sequences were trimmed and filtered following quality criteria of the processing pipeline LotuS (release 1.58) for MiSeq sequencer, as shown in the supplementary file [28]. The parameters recommended by LotuS have been used for sequencing with MiSeq. The sequences that do not meet an average quality higher than Q27 were filtered and trimmed for those reads (20 bp) with a quality lower than Q25. The final sequences less than 170 bps were discarded, obtaining the same length (170 bps). This pipeline includes UPARSE *de novo* sequence clustering and removal of chimeric sequences and phix contaminants for the identification of Operational Taxonomic Units (OTUs) and their abundance matrix generation [28,29,30]. OTU refers to organisms clustered by similarities in DNA sequence, with a sequence similarity threshold of 97% in this case [31]. Finally, taxonomy was assigned using BLAST and HITdb, a reference database for human intestinal 16S rRNA sequences, achieving up to species-level sensitivity [32]. Richness indices, diversity and non-metric multidimensional scaling (NMDS) of the OTU matrix based on the Bray-Curtis distance were calculated using raw counts and phyloseq R package [33]. The abundance matrices were then filtered and normalized in R/Bioconductor at each classification level: OTU, species, genus, family, order, class and phylum [34]. All sequencing data have been submitted to the NCBI SRA repository under the accession number PRJNA623853.

### 2.6. SNP Selection and Genotyping

A total of 95 genetic variants related to obesity and weight loss as well as interactions with dietary prescriptions were analyzed after an exhaustive bibliographic review following PRISMA criteria [35,36,37,38], whose genomic characteristics are presented. Oral epithelium samples were collected with a liquid-based kit (ORAcollect-DNA, OCR-100, DNA Genotek Inc, Ottawa, ON, Canada). Genomic DNA was isolated using the Maxwell^®^ 16 Buccal Swab LEV DNA Purification Kit (Promega Corp, Madison, WI, USA). Genotyping was performed by targeted next generation sequencing on Ion Torrent PGM equipment (Thermo Fisher Scientific Inc, Waltham, MA, USA) [39], as described elsewhere [40,41]. Overall, the amplicon mean size was 185 bp. Library construction was carried out using the custom-designed panel and the Ion AmpliSeq Library Kit 2.0 (Thermo Fisher Scientific) as per the manufacturer’s protocol. The raw data were processed with the Ion Torrent Suite Server Version 5.0.4 (Thermo Fisher Scientific Inc, Waltham, MA, USA) using Homo sapiens (genome assembly Hg 19) as the reference genome for the alignment. A custom-designed Bed file was used to locate the SNPs of interest. Variants were identified with the Torrent Variant Caller 5.0 (Thermo Fisher Scientific) with a minimum coverage value of 20. Genetic tests including Hardy-Weinberg equilibrium, linkage disequilibrium, and haplotype inferences were estimated using the Convert program (Version 1.31) and the Arlequin software (Version 3.0).

### 2.7. Genetic Risk Score (GRS)

The construction of the GRS was based on 95 SNPs previously associated with obesity in published literature. Additional information about these obesity-related SPNs can be found in previously reports [42] and Table S1 in supplementary materials. In order to confirm the association of these SNPs with BMI in Obekit population, the following steps were carried out. Firstly, Kruskal-Wallis tests were performed to identify SNPs statistically or marginally associated with baseline BMI (absence of allele, presence of one allele or presence of two alleles) in our samples, obtaining a total of 17 SNPs with a *p* value lower than 0.20. Secondly, *post hoc* tests (Mann-Whitney U test pairwise) were run to define differences between genotypes in order to be differentially coded as risk and non-risk groups in these 17 SNPs. A risk genotype was defined as the one that was associated with higher values of BMI. Genotypes with similar effects were clustered in a single category. In a third step, Mann-Whitney U test was applied to confirm statistical differences between the categorized genotype groups (risk vs. non-risk), selecting those SNPs showing at least a marginal statistical trend (*p* < 0.10) and excluding those with low sample (<10%) in either category or due to collinearity. To evaluate the combined effects of the previously selected SNPs on baseline BMI, the GRS was calculated by summing of the number of risk alleles at each locus [43], meaning that, e.g., an individual with 6 points has risk alleles for 6 out of the 10 SNPs included in this GRS. 

### 2.8. Statistical Analyses

Results were expressed as means ± standard error of the mean (quantitative variables) and as numbers and percentages (qualitative variables). The normality of analyzed variables was screened with the Shapiro-Wilk test. Statistical differences in baseline characteristics between men and women and between normal weight and overweight+obese participants were assessed by Student’s t-test or Wilcoxon rank-sum test depending on the distribution of data.

Population was divided into two groups of BMI (normalweight and overweight+obese) according to the World Health Organization criteria [20] and two groups of GRS (high and low genetic risk score split according to the mean of the population in order to obtain a similar number of subjects in each group for the statistical analyses). Linear discriminant analysis (LDA) effect size (LEfSe) (http://huttenhower.sph.harvard.edu/galaxy/) was used to compare groups and visualize the results using taxonomic bar charts. Zero-inflated Gaussian (metagenomeSeq) analysis was for finding families that differed significantly in abundance between normal-weight and obese subjects, using the cumulative sum scaling (CSS) normalization (https://www.microbiomeanalyst.ca/). Random forest, an ensemble learning method for classification and regression, was used to rank the importance of predictive variables related to BMI and GRS, using R 3.5.3 (https://www.R-project.org/) and Receiver Operating Characteristic curve (ROC) was performed to validate the random forest results (Figure S1, Supplementary material) [44]. Each input (feature) in random forest was given an importance score (MDA: mean decrease accuracy) based on the increase in error caused by removing that feature from the predictors. Random forest uses 500 trees and about two-third of the samples in the dataset as training set by randomly sampling with replacement and validates the selected features using the remaining “out-of-bag” samples. Thus, 70% of the samples were randomly chosen to train the classifier, and the remaining samples were used for validation. Canonical correspondence analysis were conducted by PAST 4 (https://folk.uio.no/ohammer/past/). Also, batch effect of samples was analyzed by principal component analysis based on Euclidean distances (Figure S2, Supplementary materials). Potential interactions between Prevotellaceae family and BMI were investigated with general linear regression models that introduced the corresponding interaction terms into the models, which were adjusted for age, sex, physical exercise and total energy intake using Stata 12. (StataCorp LLC, College Station, TX, USA; http://www.stata.com). The microbial data normalization was performed accordingly to the type of analysis. A *p* value of < 0.05 was considered statistically significant. 

## 3. Results

### 3.1. Baseline Characteristics of the Study Population

Baseline characteristics of the participants, separated by sex and by weight status, including age, anthropometric measures, and biochemical, dietary and clinical determinations are shown in Table 1. According to the BMI classification criteria of the World Health Organization, 18% (*n* = 64) of individuals were normal weight, 30% (*n* = 110) overweight and 52% (*n* = 186) obese [20]. Anthropometric variables were statistically different between normalweight men and women, except hip circumference. When comparing overweight+obese separating by sexes, all anthropometric variables were significantly different with the exception that BMI and dietary variables were statistically different, except fat intake. Some biochemical measurements such as total cholesterol, TSH and TNFα showed a difference between sexes in both groups. Also, physical exercise was influenced by sex, and was higher in men.

### 3.2. Genetic Risk Score (GRS)

To study the genetic risk association with BMI, of a total of 95 genotyped related-obesity SNPs, 10 SNPs were chosen because they were statistically or marginally associated with BMI in our population were used for calculating the GRS (Table 2). The genetic score was statistically different when comparing normalweight (5.6 ± 0.2) with overweight+obese (6.3 ± 0.1) subjects (*p* < 0.001), showing a higher value in subjects with higher BMI. The score was not significantly different between sexes in normalweight group (*p* = 0.737) and the same results were obtained when comparing this score between sexes in overweight+obese group (*p* = 0.232). The genetic score average did not differ between men (6.3 ± 0.1) and women without separating by BMI status (6.0 ± 0.1) (*p* = 0.186). 

### 3.3. Relationship between BMI, GRS and Gut Microbiome.

A lack of association was found between alpha-diversity (evaluated by Shannon index and Chao1) and BMI and GRS, when taking into account the whole population. However, when the population was separated by sex, Shannon diversity index of the families was negatively associated with BMI in men (rho = −0.21, *p* = 0.02), but not in women. On the other hand, Shannon diversity index of the families was positively associated with GRS in women (rho = 0.14, *p* = 0.02), but not in men. No association with age was found taking into account the whole population or separated by sex.

**Figure 1:**
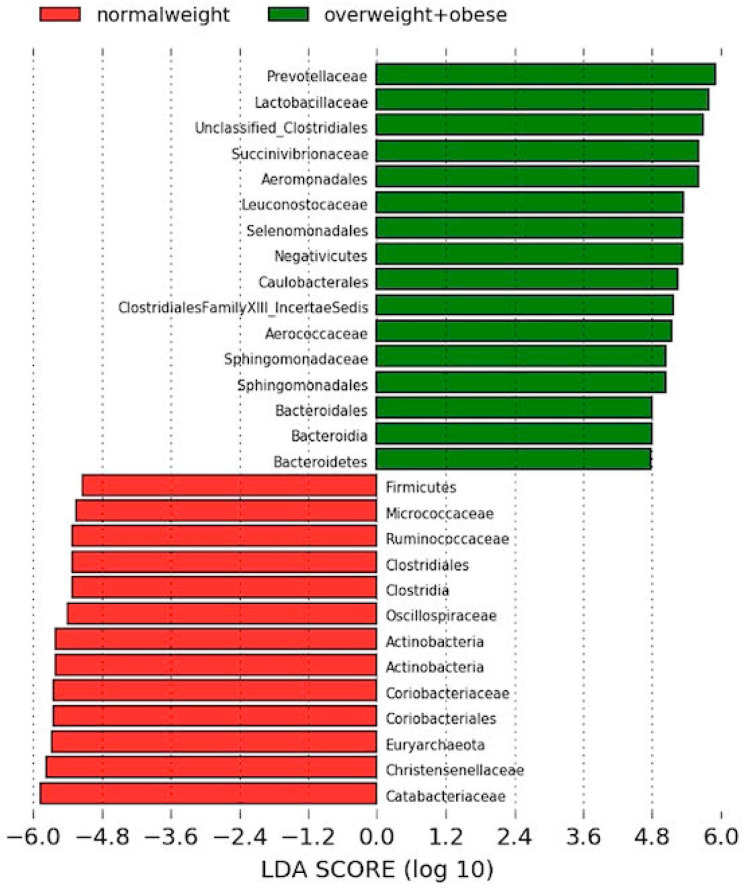
Linear discriminant analysis effect size between normal weight and overweight+obese subjects. Red, bacterial taxa statistically overrepresented in normal weight participants; green, bacterial taxa overrepresented in overweight and obese volunteers.

LEfSe analysis was used in order to evaluate microbiome differences between normal weight and overweight+obese subjects (Figure 1). Mean of abundance of these bacteria can be found in supplementary materials (Table S2).

The linear discriminant analysis effect size showed that the microbiota of the normal weight group was characterized by a preponderance of families from the Clostridiales order, such as Catabacteriaceae or Christensenellaceae. 

By contrast, the gut microbiota of the overweight+obese group gut microbiota was characterized by a preponderance of microorganisms from the Bacteroidales order (Bacteroidetes phylum), such as Prevotellaceae, and microorganisms from Bacilli class such as Lactobacillaceae (Figure 1).

**Figure 2:**
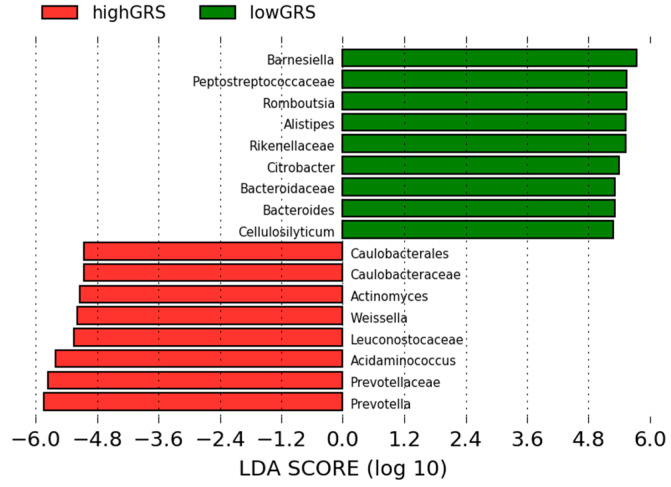
Linear discriminant analysis effect size between subjects with high Genetic Risk Score and subjects with low Genetic Risk Score Red, bacterial taxa statistically overrepresented in high Genetic Risk Score participants; green, bacterial taxa overrepresented in low Genetic Risk Score participants.

In order to evaluate whether gut microbiota differs by genetic background, we assessed the global differences of gut microbiota composition between subjects with high and low GRS by LEfSe analysis (Figure 2). Mean of abundance of these bacteria can be found in supplementary materials (Table S3).

Results showed that the Prevotellaceae family was the most predominant in subjects with high genetic risk score, including *Prevotella* genus. On the other hand, the low GRS group presented high abundance of *Barnesiella* and *Allistipes* genus (Bacteroides phylum) and Peptostreptococcaceae family from Firmicutes phylum (Figure 2). 

**Figure 3:**
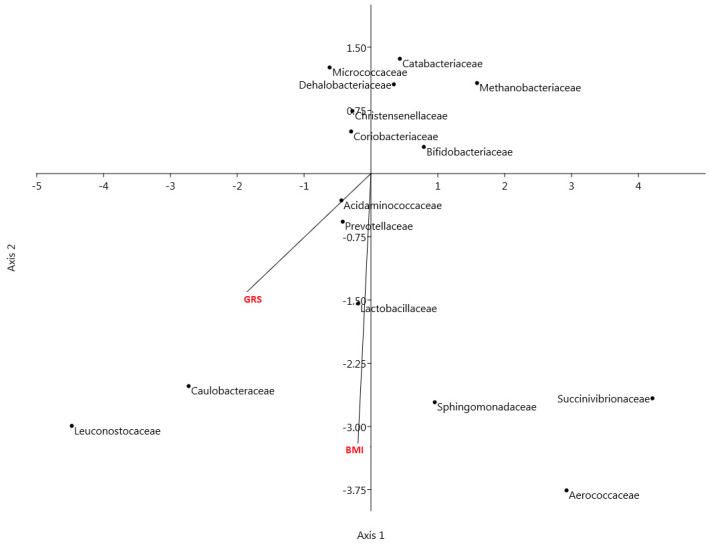
Biplot of the analysis of multivariate ordination with canonical correspondence analysis (CCA) applied to taxonomic abundance comparison at family level (including families obtained by LEfSe). CCA was performed to assess the variance in microbiota profiles at the family level in lean and overweight+obese, high GRS and low GRS and men and women. Vectors represent the environmental variables (in red) and black points represent abundance of families.

In order to visualize the relationship between bacterial families with BMI and GRS, a canonical correspondence analysis was performed. BMI and GRS were used as environmental variables with the families obtained by previous analysis. The biplot shows high correlation between BMI and GRS (Figure 3). The location of Prevotellaceae on the plot shows its association with BMI and GRS. 

**Figure 4:**
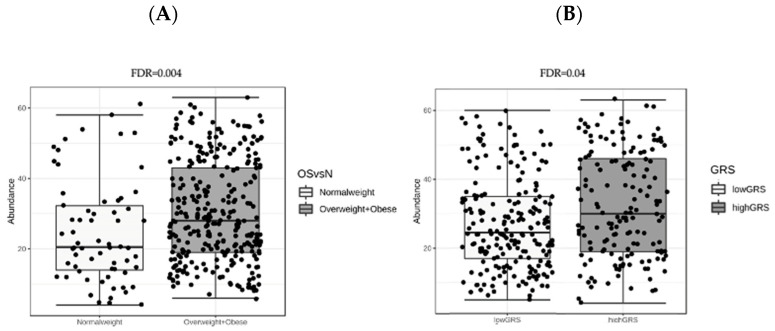
(**A**) Box plot of Prevotellaceae abundance in metagenomeSeq analysis between normal weight and overweight+obese subjects. (**B**) Box plot of Prevotellaceae abundance in metagenomeSeq analysis between low-GRS and high-GRS subjects. FDR: false discovery rate adjusted *p* value.

Moreover, metagenomeSeq analysis was performed to identify bacterial families significantly different between normal-weight and overweight+obese subjects. The figure below shows that Prevotellaceae was the only different bacterial family between groups of BMI (Figure 4A). The same analysis between high and low GRS groups showed that only Prevotellaceae family was statistically significant (Figure 4B). 

Interestingly, metagenomeSeq showed that the only significant genus was *Paraprevotella*, following by *Prevotella.* These genera are the most abundant component of Prevotellaceae family. 

**Figure 5:**
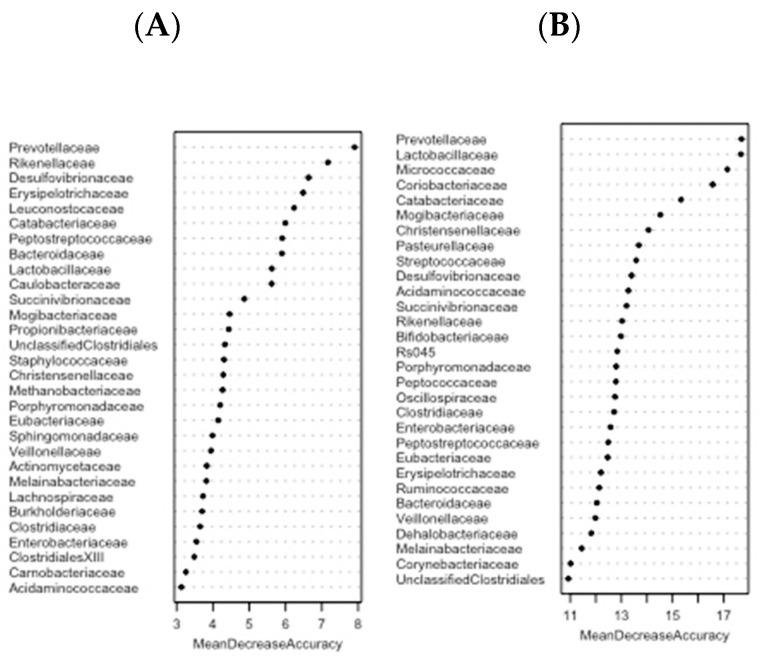
Ranking of mean decrease in accuracy (MDA) values in random forest analysis, a statistical classification that indicates the importance of each variable. Random forest calculates feature importance by removing each feature from the model and measuring the decrease in accuracy (for presence) or the increase in the mean-square error (for abundance). According to these importance scores, features were ranked in increasing order across models. The plot shows each variable on the y-axis, and their importance on the x-axis. Thus, the most important variables are at the top and an estimate of their importance is given by the position of the dot on the x-axis. (**A**) The figure shows the hierarchical rank of 30 families listed as responsible for the differences between groups of BMI. (**B**) The graph shows the hierarchical rank of importance of 30 bacterial families implicated in the differences between groups of GRS.

Furthermore, to determine the bacterial families that might be biomarkers for discriminating normalweight patients from obese, a random forest based on BMI and GRS was applied with the relative abundances of the 64 identified families included as inputs. Ten random forest models were repeated in order to rank microbial signatures that are able to differentiate groups of BMI (normalweigth and overweight/obese) or GRS (low and high) (Figure 5). To obtain a robust result, the mean of the MDA importance values was calculated for each bacterial family. Prevotellaceae showed the highest mean of importance in both approaches. 

In order to achieve the purpose of the study, to evaluate interactive associations between gut microbiota composition and polymorphisms influencing BMI, the bacterial families that presented association with both variables (BMI and GRS) in previous analyses (Prevotellaceae, Leuconostocaceae and Lactobacillaceae) were chosen to construct the linear regression models. 

**Figure 6:**
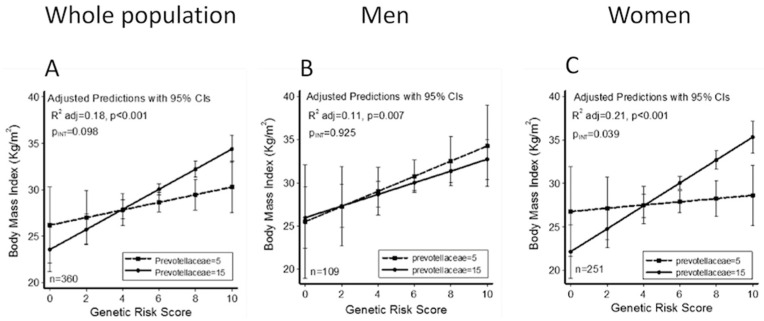
Predicted values of BMI in all population studied (**A**), men (**B**) and women (**C**) according to the Genetic Risk Score (GRS) calculated with 5 (dash line and square dots) and 15 (solid line and circle dots) relative abundance of Prevotellaceae, using linear regression models adjusted for age, sex, physical activity and energy intake, showing association between relative abundance of Prevotellaceae family and GRS.

Linear regression models adjusted for sex, age, physical exercise and energy intake were constructed for each one and the presence of interaction between microbiota and the GRS was evaluated. Only a marginal interaction between Prevotellaceae family and GRS was found (model A, Table 3). In order to evaluate the role of sex, the interaction regression models were stratified by gender (Table 3 Men and Women columns). Data revealed that a significant interaction only appeared in women (model A, *p* = 0.039) and the interaction model with Prevotellaceae and GRS improved the adjusted R^2^ value from 0.21 (Figure 6C).

## 4. Discussion

Obesity is caused by an imbalance between energy intake and energy expenditure, where complex genetic and environmental factors are involved [45,46]. Several publications have appeared in recent years documenting that genetics as well as microbiome take part in the development of obesity and others comorbidities [47,48,49]. However, the complex interactions between the intestinal microbiome, host genetics, and their mutual effects on health and disease have been scarcely studied so far. In this context, this paper is a modest contribution to the ongoing discussion about the complex relationship between gut microbiome composition and the host genotype on BMI, as well as the potential influence of the sex. 

On the one hand, the contribution of genetics to obesity has been widely recognized in humans [50]. However, most of the investigations deal with only one SNP and few of them examine the contribution of a set of several SNPs in the apparition of obesity. In this current work, a GRS construction using a set of 10 related-obesity SNPs was proposed in order to better understand the contribution of host genetics in the context of obesity, including gut microbiome composition as another important factor in this disease. It is important to underline that these SNPs have shown association with BMI in previous studies [51,52,53,54,55,56,57,58,59]. Thus, as expected, this GRS was statistically associated with BMI in our population. These findings are in line with previous studies estimating the clinical discriminative accuracy of multiple genetic testing in the prediction of common complex diseases [60,61]. Other studies have previously described different GRS in the context of obesity. Goni et al. showed that the construction of a GRS confirmed that the high genetic risk group showed greater values of adiposity than the low risk group and demonstrated that macronutrient intake modified the association between GRS and adiposity traits [62]. In the study of Hung et al., authors described a GRS with 32 well-established risk loci from a meta-analysis of Genome Wide Association Studies on BMI, which was a useful tool to predict obesity [63]. 

On the other hand, in recent years a great number of previous studies have documented several structural patterns of the gut microbial community linked to obesity, such as a high Firmicutes/Bacteroidetes ratio and low gene richness or some specific microbial families, genus and species have shown association to obesity in mice and humans [3,64,65,66,67,68]. Nevertheless, the specific members of the gut microbiota and their functional interactions contributing to obesity and associated metabolic deteriorations remain inconclusive [16]. Actually, according to the results obtained in this work, other studies that have reported a correlation between Prevotellaceae abundance and obesity. Prevotellaceae seems to be associated with an elevated level of circulating succinate concomitant with impaired glucose metabolism in obese people [69].

Therefore, a large body of data indicates a clear contribution of gut microbiota to many human diseases, but the mechanisms that mediate these associations are poorly understood, highlighting the need to better understand genetic and environmental factors affecting microbial composition. 

In this paper, particular attention was paid to how the combination of these two factors, host genetics and gut microbiome, can contribute to obesity disease. In this line, some previously research suggested that the individual microbiota composition is involved in genetically predisposed obesity. Certain studies have shown that family members have more similar microbiota than unrelated individuals [7]. For example, the concordance rate for carrying the methanogen *Methanobrevibacter smithii* is higher in monozygotic than dizygotic twin pairs [70], and some studies comparing microbiota between human subjects differing at specific genetic loci have shown gene-microbiota interactions [14,15,16,17]. In this context, Org et al. found, in a controlled environment, that the genetic background accounts for a substantial fraction of abundance of most common microbiota in mice [71,72,73,74,75]. In line with the data showed in this paper, other investigations reported a relationship between Prevotellaceae family members and host genetics. A positive correlation between *AMY1* copy number and *Prevotella* (main genus of the Prevotellaceae family) abundance highlights the role of genetics in the modulation of intestinal microbiota. In that study, authors found a positive correlation between *AMY1* copy number and gut *Prevotella* abundance, probably because this genus has enzymes and gene clusters essential for complex polysaccharide degradation and utilization [76]. Genetic variants in several genes, including *SLC9A2*, *ELAVL4* and *LINGO2*, are associated with both obesity and *Blautia* abundance, which could suggest that the mechanism of these variants acts through the gut microbiome [77]. For example, a positive association exists between a variant in *SLC9A2*, a gene that expresses a sodium/hydrogen exchanger in the colon that is upregulated in mouse models of obesity, and the abundance of the genus *Blautia* [78]. These investigations evidenced that, although it is not clear if a gut microbiota shift is the cause or the effect or obesity, the role of host genetic is this scenario is critical. Host genetics determines the metabolism and absorption of dietary nutrients, and these nutrients may influence and conditionate the presence, prevalence and the survival of a certain group of bacteria. Alike, these bacteria present important functions in the defense against foreign pathogens and the breakdown of indigestible dietary polysaccharides to produce short chain fatty acids (acetate, butyrate, and propionate), which can serve as a direct energy source for intestinal epithelial cells. Moreover, bacteria produce a wide range of other metabolites, as well as modifying human produced metabolites, such as bile acids, that can be taken up into the bloodstream where they have the potential to modulate host metabolism and other functions, even behavioral functions [79,80]. Nevertheless, in contrast to some reports in the literature, some authors consider that gut microbiome composition is shaped predominantly by environmental factors (specially the type of diet) but is not significantly associated with genetic ancestry or with individual SNPs, and, previously reported associations have not been replicated across different studies [81].

Another important issue in this complex tangle is the role of sex. In the literature, several investigations have shown sex-related differences in gut microbiota. For example, the genera *Bilophila*, *Veillonella*, and *Methanobrevibacter* were previously found to have distinct abundances in European men and women [15]. One of the most interesting approach to this issue has been proposed by Santos-Marcos et al. presenting that subjects with the same combination of metabolic syndrome criteria, showed different composition of gut microbiota depending on sex [13]. Also, the investigation of a Chinese cohort, demonstrated statistically significant differences in gut bacterial community diversity, composition, phenotypes, functions, and ecological networks, and these diverse profiles were associated with BMI but were also sex-specific [82,83,84]. 

Thus, a big number of studies show host genetic and gut microbiota are essential factors for understanding obesity; but the mechanisms by which these factors interplay with each other are poorly understood, highlighting the need to better understand genetic and environmental factors affecting microbial composition and its relationship with human health and disease. The study of the interaction between several factors, such as gut microbiota and host genetic, could explain some interindividual differences found in people with the same complex disease, such as obesity. Our finding evidenced that women with higher GRS scores and higher abundance of Prevotellaceae showed a higher BMI, while women with lower Prevotellaceae presented a lower BMI. To date, no studies have investigated the association between microbiota and a set of SNPs, and the effect on BMI. Also, other bacterial families showed an association with BMI and GRS in our research, such as Lactobacillaceae, suggesting that more research is necessary to extend our knowledge in others populations. 

Nonetheless, one limitation of this research lies in the fact that the analyses were performed at family level, and different bacterial species may be contributing to the prevalence of the family. Moreover, the problem with this approach is that the investigations concerning the influence of host genetics on the gut microbiome are difficult because gut microbiome composition can be influenced by environmental aspects such as the type of diet, physical activity or antibiotics consumption [79]. Furthermore, to determine more robust genetic associations, a larger sample size is required. However, an important strength of this investigation is the screening of multiple SNPs related to BMI, which resulted in the construction of a specific GRS. Moreover, to our knowledge, this is the first study that has evaluated the combined effects of a set of obesity-related SNP and gut microbiota composition on human obesity.

## 5. Conclusions

This proof of principle study has statistically shown that a sex-specific association occurs between host genetics and Prevotellaceae, evidencing differences in BMI according to the sex. This outcome supports the association of Prevotellaceae family with BMI, and indicates that gender differences should be taken into account.

Furthermore, the proposed GRS interacts with the Prevotellaceae family, which modulates obesity predisposition. Developing strategies to manipulate gut microbiota that drive specific components of obesity, especially those components that interact with a permissive host genetic background, may open the door to new more personalized, effective and durable approaches to prevent and treat human obesity-related diseases.

In summary, our findings should be considered as a model, which support the hypothesis that host genetics interact with the gut microbiome, and this association may play a role in obesity development, especially in women.

## Figures and Tables

**Table 1:** Baseline characteristics of the entire population and separated by sex or weight status. Values correspond to the mean ± SEM.

Variables	All Population (*n* = 360)	BMI < 24.9 kg/m^2^ Women (*n* = 46)	BMI < 24.9 kg/m^2^ Men (*n* = 18)	*p* Value ^a^	BMI > 25 kg/m^2^ Women (*n* = 205)	BMI ≥ 25 kg/m^2^ Men (*n* = 91)	*p* Value ^b^
Age (y)	44.8 ± 0.5	39.1 ± 1.3	41.5 ± 2.5	0.751	45.8 ± 0.7	46.3 ± 1.0	0.628
Weight (kg)	83.1 ± 0.8	57.8 ± 0.9	71.9 ± 1.9	<0.001	83.6 ± 0.8	97.1 ± 1.2	<0.001
BMI (kg/m^2^)	29.9 ± 0.2	21.7 ± 0.3	23.0 ± 0.3	0.009	31.6 ± 0.3	31.7 ± 0.3	0.694
Waist circumference (cm)	97.4 ± 0.8	73.1 ± 0.9	82.1 ± 1.4	<0.001	99.5 ± 0.7	108.1 ± 0.9	<0.001
Hip circumference (cm)	108.7 ± 0.6	94.3 ± 0.9	95.5 ± 1.2	0.497	113.4 ± 0.6	107.9 ± 0.7	<0.001
VAT (kg)	1.3 ± 0.1	0.2 ± 0.03	0.4 ± 0.08	0.001	1.1± 0.04	2.3 ± 0.9	<0.001
SBP (mmHg)	122 ± 1	104 ± 3	119 ± 3	<0.001	122 ± 1	132 ± 3	<0.001
DBP (mmHg)	76 ± 1	66 ± 1.9	73 ± 2.3	0.044	76 ± 1	80 ± 2	0.001
Glucose (mg/dL)	95 ± 1	83 ± 0.9	90 ± 1.3	<0.001	95 ± 1	100 ± 1	<0.001
Total cholesterol (mg/dL)	212 ± 2	191± 5.4	199 ± 6.2	0.381	215 ± 3	219 ± 4	0.407
HDL-c (mg/dL)	57 ± 1	64 ± 1.6	58 ± 2.6	0.052	59 ± 1	47 ± 1	<0.001
LDL-c (mg/dL)	136 ± 2	113 ± 5	127 ± 6	0.035	137 ± 2	147 ± 4	0.018
Triglycerides (mg/dL)	98 ± 3	68 ± 5.5	68 ± 5.1	0.555	94 ± 3	125 ± 8	<0.001
ALT (U/L)	22.6 ± 0.7	13 ± 0.4	24 ± 4.3	<0.001	21 ± 1	31 ± 1	<0.001
AST (U/L)	21.8 ± 0.4	18 ± 0.6	26 ± 1.6	<0.001	21 ± 1	25 ± 1	<0.001
Uric acid (mg/dL)	5.1 ± 0.1	3.9 ± 0.1	5.3 ± 0.2	<0.001	4.7 ± 0.1	6.0 ± 0.1	<0.001
Adiponectin (µg/mL)	11.9 ± 0.3	15.4 ± 0.7	9.6 ± 0.7	<0.001	12.8 ± 0.4	8.4 ± 0.3	<0.001
Insulin (mU/L)	7.1 ± 0.2	4.7 ± 0.3	3.5 ± 0.5	0.007	8.0 ± 0.3	8.7 ± 0.5	0.254
Leptin (ng/mL)	31.0 ± 1.6	14.2 ± 1.2	2.6 ± 0.3	<0.001	48.1 ± 1.9	14.6 ± 0.9	<0.001
CRP (µg/mL)	2.6 ± 0.2	1.5 ± 0.8	0.7 ± 0.4	0.519	2.1 ±0.2	3.2 ± 0.2	0.003
TSH (mIU/L)	1.3 ± 0.5	1.4 ± 0.1	1.3 ± 0.1	0.692	1.4 ± 0.1	1.2 ± 0.1	0.192
TNFα (pg/mL)	0.9 ± 0.1	0.8 ± 0.1	0.9 ± 0.1	0.221	1.0 ± 0.03	1.0 ± 0.03	0.258
HOMA-IR	1.7 ± 0.1	0.9 ± 0.1	0.8 ± 0.1	0.038	1.8 ± 0.1	2.2 ± 0.2	0.036
Carbohydrate intake (%)	41.1 ± 0.4	43.0 ± 1.0	44.9 ± 1.2	0.296	40.8 ± 0.5	40.4 ± 0.8	0.928
Protein intake (%)	16.9 ± 0.2	17.2 ± 0.4	15.7 ± 0.7	0.087	17.4 ± 0.2	16.0 ± 0.3	<0.001
Fat intake (%)	40.1 ± 0.3	38.3 ± 0.9	37.5 ± 1.1	0.598	40.6 ± 0.4	40.1± 0.6	0.461
Energy intake (kcal)	2907 ± 48	2560 ± 106.2	2779 ± 149.9	0.271	2849 ± 63	3237 ± 104	<0.001
Physical activity (METs)	25.3 ± 1.1	25.7 ± 2.8	47.8 ± 6.8	0.002	20.7 ± 1.2	30.9 ± 2.6	<0.001

VAT: visceral adipose tissue; SBP: systolic blood pressure; DBP: diastolic blood pressure; HDL-c: HDL cholesterol; LDL-c: LDL cholesterol; ALT: alanine aminotransferase; AST: aspartate aminotransferase; CRP: C-reactive protein; TSH: thyroid-stimulating hormone; TNFα: tumor necrosis factor alpha; HOMA-IR insulin resistance index; METs: metabolic equivalent of task. Comparisons of means between normalweight (BMI < 24.9 kg/m^2^) women and men (^a^), and overweight+obese (BMI > 25 kg/m^2^) women and men (^b^) by Student’s t-test or Mann-Whitney U test according with the distribution of data.

**Table 2:** Genotype codifications of the 10 single specific SNPs statistically or marginally associated in this population with BMI, which were used for the calculation of the Genetic Risk Score. Values correspond to the mean ± SEM.

SNP	Non-Risk Genotype	Risk Genotype	Non-Risk Group (*n*)	Risk Group (*n*)	Non-Risk Group Women BMI (kg/m^2^)	Non-Risk Group Men BMI (kg/m^2^)	Risk Group Women BMI (kg/m^2^)	Risk Group Men BMI (kg/m^2^)	*p* Value ^a^	*p* Value ^b^
rs4731426_LEP	GC	GG+CC	168	191	29.0 ± 0.5	29.5 ± 0.6	30.4 ± 0.4	31.0 ± 0.6	0.032	0.069
rs1800006_UCP3	AG	GG+AA	111	248	28.5 ± 0.5	29.8 ± 0.8	30.3 ± 0.4	30.4 ± 0.5	0.008	0.959
rs1052700_PLIN1	TT	AA+TA	46	313	28.2±0.9	28.8 ± 1.2	30.0 ± 0.3	30.5 ± 0.4	0.089	0.096
rs1042713_ADRB2	AA+AG	GG	230	129	29.5 ± 0.4	29.6 ± 0.5	30.3 ± 0.5	31.3 ± 0.7	0.217	0.034
rs11605924_CRY2	CC	AA+AC	104	255	28.7 ± 0.6	29.8 ± 0.7	30.2 ± 0.4	30.4 ± 0.5	0.024	0.455
rs1800592_UCP1	CC+CT	CT	246	113	29.3 ± 0.4	30.1 ± 0.5	30.7 ± 0.5	30.6 ± 0.8	0.092	0.321
rs2734827_UCP3	GG	AA+AG	147	212	29.1 ± 0.4	30.1 ± 0.6	30.3 ± 0.4	30.3 ± 0.5	0.039	0.714
rs1440581_PPM1K	CC	TT+TC	78	281	28.7 ± 0.8	29.6 ± 0.8	30.1 ± 0.4	30.4 ± 0.5	0.122	0.410
rs7799039_LEP	GA	GG+AA	178	178	29.3 ± 0.5	29.8 ± 0.6	30.3 ± 0.5	30.7 ± 0.6	0.101	0.223
rs12255372_TCF7L2	TT	GG+GT	51	308	28.7 ± 0.7	29.3 ± 1.1	29.9 ± 0.3	30.3 ± 0.4	0.097	0.400

Comparisons of means between non-risk women BMI and risk women BMI (^a^), and between non-risk men BMI and risk men BMI (^b^) by Mann-Whitney U test.

**Table 3:** Linear regression models constructed with BMI as dependent variable, relative abundance of Prevotellaceae family, and genetic risk score (GRS) as independent variables.

Model	Whole Population (*n* = 360)			Men (*n* = 109)			Women (*n* = 251)		
	β ± SE	*p* Value	adjR^2^	β ± SE	*p* Value	adjR^2^	β ± SE	*p* Value	adjR^2^
**Model A**		<0.001	0.18		0.007	0.11		<0.001	0.21
**Prevotellaceae**	−0.27 ± 0.25	0.276		0.004 ± 0.38	0.992		−0.44 ± 0.31	0.164	
**GRS**	0.07 ± 0.50	0.887		0.70 ± 0.80	0.383		−0.23 ± 0.63	0.713	
**Prevotellaceae#GRS**	0.06 ± 0.04	0.098		−0.006 ± 0.06	0.925		0.10 ± 0.05	0.039	
**Model B**		<0.001	0.18		0.003	0.13		<0.001	0.19
**Leuconostocaceae**	−1.37 ± 1.02	0.179		−2.12 ± 1.75	0.228		−0.17 ± 1.38	0.903	
**GRS**	0.76 ± 0.16	<0.001		0.51 ± 0.26	0.053		0.96 ± 0.21	<0.001	
**Leuconostocaceae#GRS**	0.23 ± 0.15	0.121		0.29 ± 0.28	0.305		0.08 ± 0.19	0.657	
**Model C**		<0.001	0.17		0.17	0.003		<0.001	0.19
**Lactobacillaceae**	0.42 ± 0.35	0.231		0.48 ± 0.48	0.316		0.42 ± 0.48	0.382	
**GRS**	0.99 ± 0.24	<0.001		0.79 ± 0.36	0.032		1.15 ± 0.31	<0.001	
**Lactobacillaceae#GRS**	−0.03 ± 0.05	0.506		−0.05 ± 0.07	0.511		−0.03 ± 0.07	0.648	

β represents changes in outcomes for the increasing number of units of BMI in the whole population and separating by sex; SE: standard error. Regression was adjusted for age, sex, physical exercise and energy intake excepting models stratified by sex in which the gender was not considered.

## Supplementary Materials

The following are available online at https://www.mdpi.com/2076-2607/8/6/938/s1, Figure S1: A. Roc curve BMI classification random forest for predicting obese status as variable that identified the model. B. Roc curve GRS classification random forest for predicting high genetic risk score as variable that identified the model. Figure S2: Principal component analysis (PCA) scaling based on Euclidean distances. Table S1: Genomic characteristics of the 95 obesity-predisposing SNPs. Table S2: Mean of abundance for each bacterial taxa obtained from LEfSe analysis between normalweight and overweight+obese subjects. Table S3: Mean of abundance for each bacterial taxa obtained from LEfSe analysis between high-GRS and low-GRS subjects.
