# Supplementary material for: Sex-Specific Associations between Gut Prevotellaceae and Host Genetics on Adiposity
Source: Microorganisms. 2020 Jun 22;8(6):938. doi: 10.3390/microorganisms8060938 (PMC7356943; doi:10.3390/microorganisms8060938)
Supplement: Supplementary file 1 [file microorganisms-08-00938-s001.pdf]

microorganisms

Supplementary materials

Figure S1. A. Roc curve BMI classification random forest for predicting obese status as variable that identified the model. **B**. Roc curve GRS classification random forest for predicting high genetic risk score as variable that identified the model.

A.

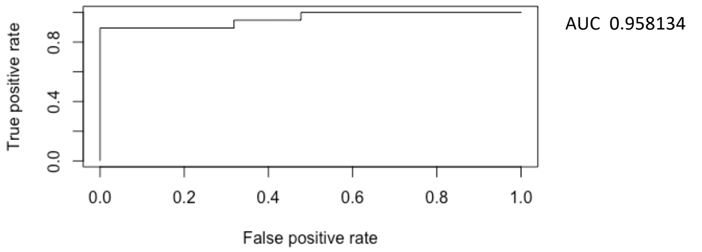

B.

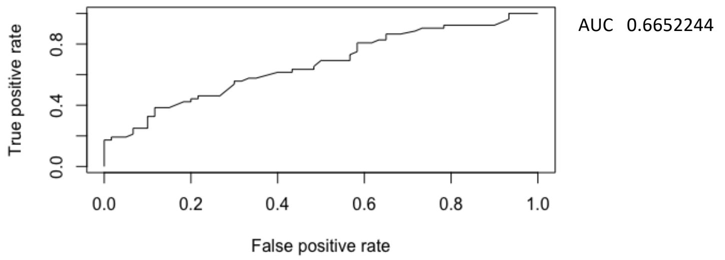

Figure S2. Principal component analysis (PCA) scaling based on Euclidean distances.

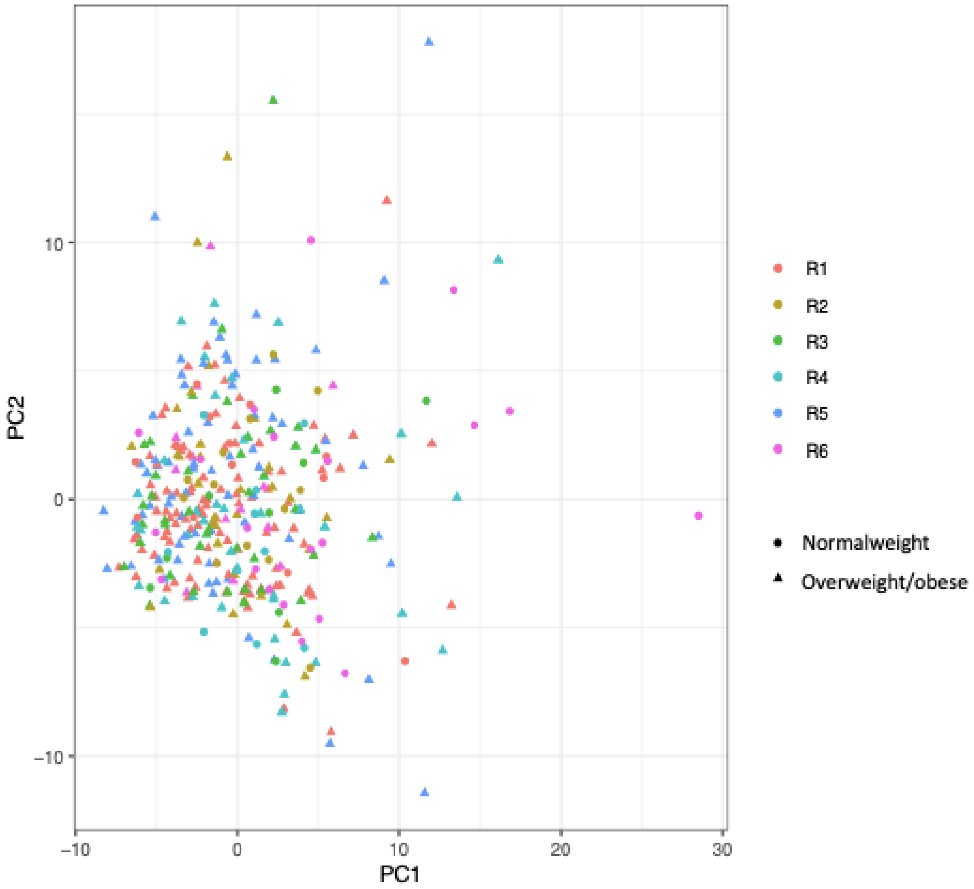

PCA plot showing the absence of batch effect. Different colors indicate different runs (R1-6) in sequencing, while different symbols represent sample types, triangle represent subjects with BMI>25kg/m^2 and circles represent subjects with BMI<24.9kg/m^2. This plot demonstrated that grouping was not caused by batch effect in our samples.

Table S1. Genomic characteristics of the 95 obesity-predisposing SNPs.

SNP[a]	Gene[b]	Chromosome position[c]	Alleles
rs17024393	*GNAT2*	Chr1:110154688	T/C
rs1801131	*MTHFR*	Chr1:11854476	T/G
rs1801133	*MTHFR*	Chr1:11856378	G/A
rs543874	*SEC16B*	Chr1:177889480	A/G
rs2605100	*LYPLAL1*	Chr1:219644224	A/G
rs4846567	*LYPLAL1*	Chr1:219750717	G/T
rs3123554	*CNR2*	Chr1:24196401	A/G
rs324420	*FAAH*	Chr1:46870761	C/A
rs8179183/rs1805094	*LEPR*	Chr1:66075952	G/C
rs2815752	*NEGR1*	Chr1:72812440	G/A
rs519887	*ABCB11*	Chr2:169780885	T/C
rs484066	*ABCB11*	Chr2:169782481	A/T
rs569805	*ABCB11*	Chr2:169782880	A/T
rs494874	*ABCB11*	Chr2:169789306	T/C
rs2943641	*IRS1*	Chr2:227093745	T/C
rs10182181	*ADCY3*	Chr2:25150296	A/G
rs713586	*ADCY3*	Chr2:25158008	T/C
rs2860323	*TMEM18*	Chr2:614210	A/G
rs2867125	*TMEM18*	Chr2:622827	T/C
rs13021737	*TMEM18*	Chr2:632348	A/G
rs1801282	*PPARG*	Chr3:12393125	C/G
rs2959272	*PPARG*	Chr3:12442833	T/G
rs1386835	*PPARG*	Chr3:12450918	A/G
rs709158	*PPARG*	Chr3:12463176	A/G
rs1175540	*PPARG*	Chr3:12465243	C/A
rs1175544	*PPARG*	Chr3:12467044	C/T
rs1797912	*PPARG*	Chr3:12470239	A/C
rs1516725	*ETV5*	Chr3:185824004	T/C
rs9816226	*ETV5*	Chr3:185834499	A/T
rs13107325	*SLC39A8*	Chr4:103188709	C/A/T
rs1799883	*FABP2*	Chr4:120241902	T/C
rs6536991	*UCP1*	Chr4:141481581	T/C
rs12502572	*UCP1*	Chr4:141485134	G/A
rs1800592	*UCP1*	Chr4:141493961	T/C
rs8192678	*PPARGC1A*	Chr4:23815662	C/T
rs10938397	*GNPDA2*	Chr4:45182527	A/G
rs1801260	*CLOCK*	Chr4:56301369	A/G
rs1440581	*PPM1K*	Chr4:89226422	T/C
rs1042713	*ADRB2*	Chr5:148206440	G/A
rs1042714	*ADRB2*	Chr5:148206473	G/C
rs6861681	*CPEB4*	Chr5:173362458	G/A
rs1800629	*TNFA*	Chr6:31543031	G/A
rs206936	*NUDT3*	Chr6:34302869	A/G
rs987237	*TFAP2B*	Chr6:50803050	A/G
rs2207139	*TFAP2B*	Chr6:50845490	A/G
rs7799039	*LEP*	Chr7:127878783	G/A
rs4731426	*LEP*	Chr7:127882070	G/C
rs2071045	*LEP*	Chr7:127892980	T/C
rs1055144	*NFE2L3*	Chr7:25871109	C/T
rs4994	*ADRB3*	Chr8:37823798	A/G
rs1800544	*ADRA2A*	Chr10:112836503	G/C
rs2419621	*ACSL5*	Chr10:114135013	C/T
rs7903146	*TCF7L2*	Chr10:114758349	C/T
rs12255372	*TCF7L2*	Chr10:114808902	G/T
rs1800497	*ANKK1*	Chr11:113270828	G/A
rs662799	*APOA5*	Chr11:116663707	G/A
rs6265	*BDNF*	Chr11:27679916	C/T
rs11030104	*BDNF*	Chr11:27684517	A/G
rs10767664	*BDNF*	Chr11:27725986	T/A
rs11605924	*CRY2*	Chr11:45873091	A/C

rs10838738	*MTCH2*	Chr11:47663049	A/G
rs660339	*UCP2*	Chr11:73689104	G/A
rs659366	*UCP2*	Chr11:73694754	C/T
rs2075577	*UCP3*	Chr11:73715542	G/A
rs2734827	*UCP3*	Chr11:73716277	G/A
rs1685325	*UCP3*	Chr11:73717025	T/C
rs2075576	*UCP3*	Chr11:73717121	C/T
rs1800006	*UCP3*	Chr11:73717254	A/G
rs1800849	*UCP3*	Chr11:73720165	G/A
rs4929949	*STK33*	Chr11:8604593	T/C
rs10830963	*MTNR1B*	Chr11:92708710	C/G
rs4769873	*ALOX5AP*	Chr13:31312689	C/T
rs1052700	*PLIN1*	Chr15:90208310	A/T
rs894160	*PLIN1*	Chr15:90211823	C/T
rs2289487	*PLIN1*	Chr15:90217096	C/T
rs7498665	*SH2B1*	Chr16:28883241	A/G
rs7359397	*SH2B1*	Chr16:28885659	C/T
rs1558902	*FTO*	Chr16:53803574	T/A
rs1121980	*FTO*	Chr16:53809247	G/A
rs17817449	*FTO*	Chr16:53813367	T/G
rs8050136	*FTO*	Chr16:53816275	C/A
rs3751812	*FTO*	chr16:53818460	G/T
rs9939609	*FTO*	Chr16:53820527	T/A
rs12452844	*AANAT*	Chr17:74459243	G/A
rs1805081	*NPC1*	Chr18:21140432	T/C
rs6567160	*MC4R*	Chr18:57829135	T/C
rs571312	*MC4R*	Chr18:57839769	C/A
rs17782313	*MC4R*	Chr18:57851097	T/C
rs17066866	*MC4R*	Chr18:58055619	A/T
rs17069904	*TNFRSF11A*	Chr18:60032949	G/A
rs2287019	*QPCTL*	Chr19:46202172	C/T
rs6013029	*CTNNBL1*	Chr20:36399580	G/T
rs6123837	*GNAS*	Chr20:57465571	G/A
rs3813929	*HTR2C*	ChrX:113818520	C/T
rs11091046	*AGTR2*	ChrX:115305126	A/C

[a] dbSNP reference, [b] gene assigned by dbSNP and [c] GRCh37, p13 assemb

Table S2. Mean of abundance for each bacterial taxa obtained from LEfSe analysis between normalweight and overweight+obese subjects.

TAXA NAME	**Mean of abundance in normalweight subjects**	**Mean of abundance in overweight+obese subjects**
Actinobacteria	10.6 ± 0.2	9.8 ± 0.1
Bacteroidetes	16.8 ± 0.05	16.9 ± 0.02
Firmicutes	16.1 ± 0.7	15.8 ± 0.03
Actinobacteria (class)	10.6 ± 0.2	9.7 ± 0.1
Bacteroidia	16.8 ± 0.05	17.0 ± 0.02
Clostridia	15.8 ± 0.1	15.4 ± 0.05
Negativicutes	11.4 ± 0.2	11.8 ± 0.08
Aeromonadales	0.9 ± 0.2	1.7 ± 0.2
Bacteroidales	16.8 ± 0.05	17.0 ± 0.02
Caulobacterales	0.3 ± 0.1	0.6 ± 0.06
Clostridiales	15.8 ± 0.1	15.4 ± 0.05
Coriobacteriales	9.0 ± 0.2	8.14 ± 0.09
Selenomonadales	11.4 ± 0.2	11.8 ± 0.09
Sphingomonadales	0.17 ± 0.08	0.4 ± 0.04
Aerococcaceae	0.1 ± 0.07	0.4 ± 0.05
Catabacteriaceae	5.7 ± 0.4	4.3 ± 0.2
Christensenellaceae	8.0 ± 0.4	6.9 ± 0.2
Clostrdidiales FamilyXIII_IncertaeSedis	0.06 ± 0.03	0.4 ± 0.06
Coriobacteriaceae	9.0 ± 0.2	8.1 ± 0.1
Lactobacillaceae	2.5 ± 0.3	3.7 ± 0.2
Leuconostocaceae	2.0 ± 0.2	2.4 ± 0.2
Micrococcaceae	1.9 ± 0.2	1.5 ± 0.1
Oscillospiraceae	12.9 ± 0.2	12.4 ± 0.1
Prevotellaceae	11.15 ± 0.5	12.7 ± 0.2
Ruminococcaceae	14.4 ± 0.1	14.0 ± 0.1
Sphingomonadaceae	0.2 ± 0.07	0.4 ± 0.04
Succinivibrionaceae	0.8 ± 0.2	1.7 ± 0.2
Unclassified_Clostridiales	1.8 ± 0.4	2.7 ± 0.2

Values correspond to the mean ± SEM.

Table S3. Mean of abundance for each bacterial taxa obtained from LEfSe analysis between high-GRS and low-GRS subjects.

TAXA NAME	Mean of abundance in high GRS subjects	Mean of abundance in low GRS subjects
Bacteroides	16.0 ± 0.06	16.2 ± 0.05
Caulobacterales	0.6 ± 0.08	0.4 ± 0.06
Bacteroidaceae	16.0 ± 0.06	16.2 ± 0.05
Caulobacteraceae	0.6 ± 0.08	0.4 ± 0.07
Leuconostocaceae	0.8 ± 0.1	0.5 ± 0.07
Peptostreptococcaceae	8.0 ± 0.1	8.4 ± 0.1
Prevotellaceae	13.0 ± 0.3	12.0 ± 0.2
Rikenellaceae	12.1 ± 0.1	12.5 ± 0.1
Acidaminococcus	3.5 ± 0.3	2.8 ± 0.2
Actinomyces	1.5 ± 0.1	1.3 ± 0.09
Alistipes	12.1 ± 0.1	12.5 ± 0.1
Barnesiella	6.4 ± 0.3	7.3 ± 0.2
Celluslostiycum	0.09 ± 0.04	0.5 ± 0.1
Citrobacter	0.6 ± 0.1	1.0 ± 0.2
Prevotella	11.8 ± 0.3	10.5 ± 0.3
Romboutsia	8.0 ± 0.1	8.4 ± 0.1
Weisella	0.6 ± 0.1	0.3 ± 0.07

Values correspond to the mean ± SEM.
